# Combination of Multimodal MRI, Neuronavigation, and Awake Craniotomy in Removing Tumors of Eloquent Areas

**DOI:** 10.17691/stm2022.14.2.06

**Published:** 2022-03-28

**Authors:** А.S. Zolotova, М.S. Evstigneyev, К.S. Yashin, А.Yu. Ermolayev, М.V. Ostapyuk, V.M.A. Al-Madhadjy, V.I. Zagrekov, N.Yu. Antonova, М.V. Shibanova, L.Ya. Kravets, N.E. Gronskaya, I.А. Medyanik

**Affiliations:** Staff Physician, Department of Oncology and Neurosurgery, University Clinic; Privolzhsky Research Medical University, 10/1 Minin and Pozharsky Square, Nizhny Novgorod, 603005, Russia;; Anesthesiologist-Resuscitator, University Clinic; Privolzhsky Research Medical University, 10/1 Minin and Pozharsky Square, Nizhny Novgorod, 603005, Russia;; Neurosurgeon, University Clinic; Privolzhsky Research Medical University, 10/1 Minin and Pozharsky Square, Nizhny Novgorod, 603005, Russia; Assistant, Department of Traumatology, Orthopedics, and Neurosurgery; Privolzhsky Research Medical University, 10/1 Minin and Pozharsky Square, Nizhny Novgorod, 603005, Russia;; Neurosurgeon, University Clinic; Privolzhsky Research Medical University, 10/1 Minin and Pozharsky Square, Nizhny Novgorod, 603005, Russia;; PhD Student, Department of Traumatology, Orthopedics, and Neurosurgery; Privolzhsky Research Medical University, 10/1 Minin and Pozharsky Square, Nizhny Novgorod, 603005, Russia;; Staff Physician, Department of Oncology and Neurosurgery, University Clinic; Privolzhsky Research Medical University, 10/1 Minin and Pozharsky Square, Nizhny Novgorod, 603005, Russia;; Head of the Department of Anesthesiology and Resuscitation, University Clinic; Privolzhsky Research Medical University, 10/1 Minin and Pozharsky Square, Nizhny Novgorod, 603005, Russia;; Intern Researcher; National Research University Higher School of Economics, 25/12 Bolshaya Pecherskaya St., Nizhny Novgorod, 603155, Russia; Student; National Research University Higher School of Economics, 25/12 Bolshaya Pecherskaya St., Nizhny Novgorod, 603155, Russia; Professor, Chief Researcher of the Neurosurgery Group, University Clinic; Privolzhsky Research Medical University, 10/1 Minin and Pozharsky Square, Nizhny Novgorod, 603005, Russia;; Director of the Language and Brain Center; National Research University Higher School of Economics, 25/12 Bolshaya Pecherskaya St., Nizhny Novgorod, 603155, Russia; Neurosurgeon; Privolzhsky Research Medical University, 10/1 Minin and Pozharsky Square, Nizhny Novgorod, 603005, Russia;

**Keywords:** awake craniotomy, intracerebral tumors, eloquent areas, multimodal MRI, neuronavigation

## Abstract

**The aim of the study** is to assess the possibilities of the combined approach to using multimodal MRI, neuronavigation, and awake craniotomy in resecting tumors of eloquent areas.

**Materials and Methods:**

The results of 30 successive awake surgical interventions performed in 2017−2019 years in patients with tumors of eloquent areas have been analyzed. The main selection criterion for this type of operations was the location of the tumor in the projection or in the immediate proximity to the cortical centers of speech and motion. To minimize the damage, patients underwent functional MRI and DTI tractography at the prehospital stage to identify cortical regions and white matter tracts involved in the motor and language functions; immediately before the operation the acquired data was loaded into the navigation StealthStation S7 (Medtronic, USA) to plan and monitor surgery stages; during the surgery, direct cortical and subcortical stimulation was performed to identify the motor and speech centers (asleep−awake−asleep technique) with neurolinguistic testing. Karnofsky performance status, assessment of the patient’s neurological status, frequency of epileptic seizures before and after the operation, the extent of the tumor resection, and the data analysis after the linguistic testing were used to determine the patients’ condition and surgery outcomes.

**Results:**

Improvement of the general state after the operation has been noted in 30% of patients compared to the preoperative condition, no neurological deficit dynamics has been observed in 33% of patients. Postoperative multimodal MRI showed that total tumor removal was achieved in 37% of cases, subtotal in 40%, partial removal resection in 23% of cases.

**Conclusion:**

The combined approach to the brain tumor resection using multimodal MRI, neuronavigation, and awake craniotomy with motor and language areas mapping allows neurosurgeons to minimize the risk of persistent neurological deficit occurrence and provides the possibility to perform maximal resection possible preserving the patients’ functional status. The presented methodology is reproducible, permitting one to expand the options of surgical treatment when lesions are localized in eloquent areas.

## Introduction

Tumors localized in or near eloquent brain regions represent a serious surgical problem. Tumor resection close to these regions is associated with a high risk of persistent neurological deficit. Thus, the resection extent correlates with the survival in patients with gliomas of various malignancy grades [1-3].

The main goal of the surgery on such eloquent brain areas is to perform maximal resection possible while preserving patient’s functional status. Zones responsible for motion, speech, and cognitive skills play the decisive role in the occurrence of the persistent neurological deficit, negatively affecting the subsequent course of the disease and patient survival [4-6].

In order to preserve the functional status of a patient, it is important to establish the relationship between the tumor and the motor and speech cortical centers and the tracts of the brain white matter at the preoperative stage. The multimodal MRI capabilities are actively used nowadays to fulfill this task. Functional mapping including functional MRI (fMRI) and diffusion tensor imaging (DTI tractography) is usually conducted [7]. For intraoperative control, direct cortical and subcortical stimulation is carried out with the functional status control of the awake patient [8, 9].

The proposed combined approach to the tactics of surgical tumor treatment of the eloquent brain areas comprising fMRI, DTI tractography, and awake craniotomy gives a number of advantages: lower risks of persistent neurological deficit, the feasibility of more extended resection, and preservation of the patient’s functional status using intraoperative monitoring.

**The aim of the study** is to assess the results of the combined approach to the resection of eloquent areas.

## Materials and Methods

### Patients

In the Neurosurgical Department of the University Clinic of Privolzhsky Research Medical University (Nizhny Novgorod, Russia), 30 awake brain tumor resections were performed in 2017–2019 years. The study included 29 patients (14 women, 15 men); of them, one patient underwent the procedure twice with a 4-month interval. The average age was 41 [37; 51] years.

Patients were selected for this manipulation according to the following criteria: 1) the location of the tumor in the projection or in the immediate proximity to the cortical centers of speech and motion; 2) absence of the excessive neurological deficit, which may interfere with the test performance (evident aphasic disorders, motor deficit); 3) patient’s consent.

Claustrophobia, uncontrolled cough, very young age, pathologic obesity, chronic diseases, marked brain edema were considered as relative contra-indications.

The preoperative neurological status analysis has shown presence of epileptic seizures before the operation in 47% of cases (14/30), the mean value of the Karnofsky perfomance score (KPS) was 80 [70; 90] points. Focal neurological deficit prior to the operation was observed in 27% of cases (8/30), consisting of only aphasic (10%), only cognitive (6.7%), or aphasic and motor (10%) disorders.

The most prevailing tumors were grade III (13 patients) and grade II gliomas (6 patients). Grade IV glioblastoma was verified in 2 patients, grade I glioma in 5 patients. Four patients with metastatic brain lesion were also operated on. The tumor volume determined by MRI was 62.25 [26.30; 101.0] cm^3^. In 15 patients, the tumor involved 2 and more brain lobes.

Of 30 operations done, 21 were performed for the first time, 9 operations after surgical (n=1) or combined (n=8) treatment. Prior to the operation, patients were informed about the forthcoming intervention, its stages, the necessity to tell the surgeon or anesthesiologist about worsening of the state, feeling of nausea, pain, or numbness in any part of the body.

The study was done in compliance with the Declaration of Helsinki (2013). Written informed consent was obtained from each patient. The work was approved by the Ethics Committee of Privolzhsky Research Medical University.

### Multimodal MRI

All patients underwent MRI examination using Magnetom Essenza 1.5 Т system (Siemens, Germany) before the operation. The protocol of preoperative MRI examination included the following modes: T2-WI; FLAIR in the axial plane; T1-WI MPR iso (1 mm); DTI (64 diffusion directions; b-value — 1000 s/mm^2^; voxel size — 3×3×3 mm); BOLD (with presentation of paradigms for passive speech, active speech, arm motion, leg motion). Post-processing was done using the software package Nordic Neurolab (Norway). It included MR-tractography with the reconstruction of the main pathways of the damaged hemisphere (mesolobus, cortico-spinal tracts, radiate crown, superior and inferior longitudinal fasticuli, inferior fronto-occipital fasciculus, uncinate, arcuate fasticuli, frontal aslant tract) and building a map of brain activity in compliance with the mentioned paradigms. The obtained MRI data were loaded to the navigation station StealthStation S7 (Medtronic, USA) to plan the stages of the operative intervention.

### Preoperative speech testing

Each patient underwent neurolinguistic testing which consisted of preoperative testing to define a general speech status of the patient; selection and conduction of intraoperative tests corresponding to the tumor location; intraoperative linguistic speech mapping; postoperative testing in order to reveal changes in the speech. The Russian Aphasia Test (RAT) was used for this purpose [10]. This battery of tests was composed of 13 subtests aimed at the assessment of the ability to production, comprehension, and repetition of the speech and is valid and reliable for tracing the changes in the speech at each linguistic level. Testing was carried out 1–2 days prior to the operation to form an individual battery of test tasks. Test samples with mistakes were excluded from the results. This stage was necessary in order to present only those stimuli to the patient during intraoperative testing which had not caused difficulties for minimizing the risk of mistakes not related to electrical stimulation.

### Intraoperative mapping of the speech eloquent areas

The neurolinguistic testing could include 1, 2, or 3 tests depending on the tumor location. Thus, to map the functions of the frontal lobe and underlying pathways (frontal aslant tract and anterior parts of the arcuate fasciculus), tasks involving naming actions and finishing sentences were used [11]. Tasks involving naming objects and phonologic discrimination were employed for mapping the temporal, occipital, and underlying pathways (arcuate fasciculus — short and dorsal segments, superior longitudinal fasciculus, inferior longitudinal fasciculus, fronto-occipital fasciculus) [12]. If a speech deficit was detected the corresponding area was labeled with a sterile mark.

### Cortical and subcortical stimulation

ISIS IOM system (inomed Medizintechnik GmbH, Germany) was used for neurophysiological monitoring. The zone of electrode action was chosen on the basis of neuronavigation data. This zone covered the region of the pathological focus and adjacent areas of the cortex at a distance not less than 1–2 cm from the tumor margins. Bipolar electrode supplying two-phase current, the intensity of which was set within the standard values of 6–12 mA, was used for cortical mapping. For subcortical mapping, a 1–20-mA monopolar electrode was applied.

To map the cortical representations of the motor function, current stimulation was done with the registration of the motor response on the contralateral side with subcutaneous needle electrodes.

### Operative intervention

Operations were performed using a microsurgical technique. The area of trepanation and skin incision was planned with the help of the neuronavigation system, into which data of multimodal MRI examination were loaded.

Locoregional anesthesia of the nerve branches innervating the scalp was conducted to each patient to prevent pain sensation after awakening. The skin at the site of the Mayfield clamp fixation and incision zone were also anesthetized. 0.375% ropivacaine with adrenaline in the ratio 1:200,000 and 40–60-ml volume were used for the blockage. After the induction anesthesia and trachea intubation, anesthesia was maintained by continuous infusion of propofol (20–40 ml/h) and fentanyl (0.5 ml/h).

After patient’s awakening, the neurosurgeon established a contact with the patient and started cortical stimulation. Simultaneously, the patient was tested by a neurolinguist. During tumor removal, resection was interleaved with electrostimulation in order to control function preservation. Seizures occurring during stimulation were arrested with an ice-cold sterile Ringer’s solution with subsequent reduction of the current intensity. Resection continued up to the visually unchanged brain matter, and when the response to the stimulation was received at 1–2-mА current (which corresponds to the distance of 1–2 mm), the resection was terminated. After the completion of the main stage, sedation of the patient was conducted with dexmedetomidine or propofol with midazolam depending on the clinical necessity.

All operations were monitored by neuronavigation and additional methods of intraoperative neurovisualization were also employed: ultrasound control in 10 cases (33%), optical coherence tomography in 4 cases (13%).

After the operation, patients were supervised by resuscitators and if there were no contraindications, they were transferred to the Department of Neurosurgery.

30 awake craniotomies have been performed; in one case, the procedure had to be discontinued as neurolinguistic testing appeared to be impossible due to hyper reaction of the patient after waking up. The patient was reanesthetized and the operation was continued under general anesthesia.

### Assessment of resection radicality and treatment results

Control MRI examination and repeated neurolinguistic testing were conducted in the postoperative period. The extent of resection was determined quantitatively by calculation of the ratio of the residual to the preoperative tumor volume using manual segmentation of the zone of pathological T2/ FLAIR signal for non-contrast-enhanced gliomas and the zone of contrast agent accumulation for metastases and contrast-enhanced gliomas.

Segmentation and calculation of the tumor volume were done with the help of the navigation StealthStation S7. The removal was considered total if the resection rate was more than 99% (insignificant accumulation of the contrast reinforcement on the walls of the postoperative cyst was permitted), subtotal if radicality amounted to 70– 99%, and partial at radicality below 70%.

The KPS, epileptic seizure rate before and after the operation, the analysis of pre- and postoperative neurological deficits, repeated linguistic testing served to indicate the general state of the patient.

### Statistical data processing

Statistica 10.0 software package was used for statistical data analysis. Since the number of patients in the general population was below 50 and the numerical values in the sample were not normally distributed, nonparametric methods of statistical analysis have been applied. To analyze the changes of the parameters in the process of the study, Wilcoxon signed-rank test was used, and p<0.05 was considered to be the lower border of significance. Numerical data were presented as median (Me) and interquartile range [Q1; Q3].

## Results

### Duration of the operarion and hospital stay

The length of the preparatory stage including determination of the optimal access with neuronavigation took on average 85 [75; 105] min. The access occupied about 60 [50; 65] min. The mean duration of the main operative stage was 220 [180; 250] min.

Having the maximally possible tumor volume removed, the final stage of the operation, lasting 85 [65; 95] min on average, was performed under general anesthesia. The average volume of blood loss amounted to 150 [100; 200] ml.

The average hospital stay was 8.5 [7.0; 11.0] days.

### Results of cortical mapping

During cortical stimulation, positive testing result was determined in 70% of cases; in subcortical, in 30% of cases (stimulation is positive if it reveals a deficit).

In 7% (2/30) of cases, epileptic seizures were observed: generalized and partial simple ones. None of them required termination of the awake phase and was arrested with the above-mentioned methods.

### Dynamics of neurological deficit

In the postoperative period, epileptic seizures occurred in 17% of cases (5/30); in two patients, they were registered for the first time. Increase of neurological deficit after the operation was noted in 38% of cases (11/30): in 17% (5/30), aphatic disorders were diagnosed; in 10% (3/30), they were motor; in 3% (1/30) — cognitive, and in 7% of cases (2/30) both motor and aphatic disorders were found. Complete regress of neurological symptoms was noted in 13% of cases (4/30) by the time of discharge.

The KPS after the operation was 80 [70; 90] points. Improvement of the general condition after the operation was noted in 30% of cases (9/30), absence of neurological deficit dynamics was observed in 33% of cases (10/30). General data on the patients’ state in the postoperative period is presented in the Table.

**Table:** Characteristics of the patients in postoperative period

Parameters	number Abs.	%
Karnofsky performance status (points)	80 [70; 90]	
Epileptic seizures	5	17
Infectious complications	1	3
Hemorrhagic complications	1	3
Neurological deficit:		
immediately after the operation	11	37
by discharge	7	23
Neurological symptoms regress in dynamics	4	13
Improvement of general state	9	30
Absence of dynamics in patients’ state	10	33

According to the RAT before and after the operation, no statistically significant difference was obtained in the intensity of aphatic disorders: 9.8 [9.4; 9.9] points before the operation, 9.75 [9.10; 10.0] points after it; p=0.67.

### Radicality of tumor resection

The postoperative MRI examination showed that the total tumor removal was achieved in 11 patients (37%), subtotal in 12 patients (40%). Partial removal was conducted in 7 patients (23%).

## Discussion

Radical resection improves general survival in glial tumors of various degrees of malignancy. But preservation of patients’ quality of life and functional status is no less important. Location of cortical centers for motor, sensory, and speech areas varies in different patients causing a high risk of neurological disorders during tumor resection, especially in case of indistinct visually defined borders between a healthy and tumorous tissue [13].

Direct cortical and subcortical stimulations are the basic methods of defining eloquent areas. Preoperative mapping by means of noninvasive methods such as multimodal MRI and DTI tractography is also actively being used in the neurosurgical practice. Moreover, each methodology has its own advantages and restrictions on use.

According to the investigations made by Roux et al. [14], the sensitivity of fMRI is 59–98%, while specificity makes up 97–98%. These wide ranges are explained by heterogeneity of the language stimuli and visualization protocols [14, 15]. One of the restrictions on the use of fMRI is the effect of the obtained results of biological aspects of the tumor and the patient’s functional state [16, 17]. The data analysis presented in the work by Kapsalakis et al. [18] has shown that patients with a better functional state demonstrated a higher compliance with the data of fMRI and direct cortical stimulation. Correlation was also found between the degree of tumor malignancy and frequency of data compliance of the two methods.

Investigating the accuracy of fMRI relative to the direct cortical stimulation, the authors [19] came to the conclusion that usage of fMRI only is a less reliable method. Other authors in the similar study [20] have stated that fMRI data are sufficiently accurate for the preoperative surgical planning and determination of the necessity of intraoperative direct cortical mapping. Moreover, fMRI is a useful supplement to the direct cortical stimulation since the data loaded into the neuronavigation system help in the selection of the place for electrode installation [18, 20].

The direct cortical stimulation has also its restriction: unavailability for preoperative planning, risks of intraoperative epileptic seizures and transition to general anesthesia, increased total duration of the operation, and a psychological load for the patient and the team [21].

In the course of this study, the frequency of the transition to general anesthesia was 3% due to the absence of the productive contact with the patient. According to the meta-analysis data [22], the transition toward the general anesthesia makes up 2% and does not actually depend on the anesthesia protocol used (asleep–awake–asleep/monitored anaesthesia сare/ awake–awake–awake). To reduce the risks of this complication, it is necessary to make a careful selection of patients and give them more ample information about the forthcoming procedure.

It is important to note that in this study, the neurosurgeon continued the operation after the transition to general anesthesia using the data of fMRI and DTI tractography. In their work, Rigolo et al. [17] compare two groups of patients: direct cortical stimulation was successively performed to the first group; stimulation could not be done to the second group and the operation was continued using fMRI data. The authors report that there was no significant difference in the rate of postoperative deficit between these two groups. This demonstrates additional value of fMRI beyond the preoperative planning.

The main factors limiting the application of the cortical and subcortical stimulation are intraoperative epileptic seizures and inability to establish a contact with the patient after awakening. According to the works [23-25], intraoperative seizures arise in 2.2–21.9% of awake craniotomy cases. This difference in values may be accounted for by diverse protocols of cortical stimulation. Szelényi et al. [26] believe that intraoperative seizures correlate with stimulus duration, current intensity, and the number of repeated stimulations of one and the same area. In this study, the rate of epileptic seizures was reported to be 7% (in 2 patients), stimulation lasted for less than 3 s, verification of the functional zone was done from 1 to 3 times, without the repeated stimulation of the zone, at 1–20-mA current. Intraoperative epileptic seizures may be the cause of the termination of the awake stage or the entire operation [27, 28]. Nossek et al. [28] reported 12.6% of the seizure rate and in 18% of these cases, the operation had to be terminated. In our study, none of the operations was stopped due to the emerging seizure, and after its arrest, in one case, the patient was able to continue testing. Following the protocol of electric stimulation and timely arresting the seizures minimize possible intraoperative risks.

Patient’s awakening and neurolinguistic tests increase the duration of the operation. The average time of the main stage of the surgical interventions was 205 [90; 300] min as described in the article written by Joswig et al. [29], in which they highlighted the results of the first experience in performing awake craniotomy, and this result is almost similar to our data. In this case, the duration of the operation depends on various factors including a coordinated work of the surgery team, individual sensitivity of the patient to anesthesia, and intraoperative complications.

The percentage of “positive” stimulation in our study was 78 and 40% for cortical and subcortical stimulation, respectively. Data of other authors differ: some hold to the concept of positive mapping, others to the negative mapping. Today, positive mapping, i.e. identification of at least one speech zone, is accepted as a standard intraoperative method of preserving the functional status of the patient [15]. However, it is not always that the speech zone can be found or the search may be long enough, therefore some authors believe that under the conditions of the limited cortical action, negative mapping allows for a more aggressive glioma resection [6]. This strategy has some advantages such as minimal cortical impact, less extensive intraoperative mapping, and as a consequence, shorter duration of the operation [26]. The question concerning the effectiveness of the negative mapping, i.e. whether this method influences the rate of persistent neurological deficit, remains open. Sanai et al. [30] have shown in their study that only 1% of patient developed persistent aphatic disorders 6 months after the operation, although the speech zones were not identified in half of the patients. However, Duffau [31] believes that the negative mapping method may be used only in relation to highly differentiated gliomas, since the aim of surgical intervention is to resect as much of the tumor as possible.

DTI tractography may be employed at the preoperative stage to assess the effect of the tumorous tissue on the white matter fibers, identify its resectability, and for surgical intervention planning [32]. Besides, 3D reconstruction of the acquired data loaded into the neuronavigation system may help in complete tumor resection. And, in combination with the direct cortical stimulation, tractography promotes the reduction of patient’s wake time and the number of electric stimuli owing to the less time spent for detecting functional zones [33, 34]. In the review on this topic, Vanderweyen et al. [33] distinguish two groups of factors restricting the application of this method. The external restrictions are the brain shift and peritumoral edema. The combination of intraoperative methods (e.g. proposed in the present study: fMRI and direct cortical stimulation) helps achieve better outcomes. All difficulties connected with the acquisition and analysis of data were referred by the authors to the internal restrictions, which are solved by improvement of the technical tools within the structure of the developing direction called connectomics [34].

The choice of the tactics for surgical treatment of tumors of the eloquent areas in favor of the combined approach including multimodal MRI, DTI tractography, and cortical stimulation in awake patients gives a number of advantages.

1. This combination of methods ensures the reduction of risks of persistent neurological deficit. In our study, a postoperative deficit was observed on the first day in 37% of cases, by the time of the discharge the deficit persisted in 23% of cases. According to the foreign sources [29, 35], early neurological deficit in patients after awake craniotomy occurred in 29–30%, which is comparable with our results. In the study conducted by Kumar et al. [7], where a similar combined method was used, early neurological deficit was determined in 26%, whereas persistent neurological deficit was found only in 6% of patients 6 months after the operation.
2. The combination of stated methods makes it possible to compensate for the limitations of each of them. Thus, Rolinski et al. [36] supposed that fMRI may be more sensitive and less specific than direct cortical stimulation, but their combined application helps prevent possible deficit better than any method taken separately.
3. The multimodal approach allows the surgeon to perform a more extended resection. In our study, we succeeded in making the total tumor removal without any increase of neurological deficit in 37% of cases. Investigating the effect of different methods on the extent of resection of highly differentiated gliomas, Incekara et al. [37] came to the conclusion that direct cortical stimulation in combination with fMRI and DTI tractography improved the resection extent considerably especially in comparison with the cases where only the neuronavigation system was used.

## Conclusion

The combined approach to the removal of tumors in the eloquent areas using multimodal MRI, neuronavigation, and awake craniotomy with mapping of the speech and motor zones allows neurosurgeons to minimize the risk of persistent neurological deficit and provides the possibility to perform maximal resection possible preserving the functional status of the patient. The presented methodology is reproducible, permitting one to expand the options of surgical treatment when lesions are localized in the eloquent areas.
